# Obstructing primary squamous cell carcinoma of caecum: A case report

**DOI:** 10.1016/j.amsu.2022.103907

**Published:** 2022-06-06

**Authors:** Bhabani Sankar Sahoo, Sri Aurobindo Prasad Das, Sonia Badwal, Samiran Nundy, Naimish N. Mehta

**Affiliations:** a Department of Surgical Gastroenterology and Liver Transplantation, Sir Ganga Ram Hospital, India; b Department of Pathology, Sir Ganga Ram Hospital, India

**Keywords:** SCC, Squamous Cell Carcinoma, CEA, Carcino Embryonic Antigen, FDG-PET, Fluorodeoxy Glucose-Positron Emission Tomography, POD, Post Operative Day, AJCC, American Joint Committee on Cancer, HPV, Human Papilloma Virus

## Abstract

Squamous cell carcinoma (SCC) of the colon is a rare malignancy and usually a pathological surprise. Clinical presentation is usually very similar to adeno carcinoma variety. We report a case of a 56 year old male with primary SCC of caecum presenting as small bowel obstruction and managed with surgery and adjuvant chemotherapy. It was labelled as primary SCC after extensive search for other primary malignant SCC in body with possible metastasis to caecum. Due to rarity of the disease and lack of literature standardized protocols for neo-adjuvant and adjuvant therapy are not available.

## Highlights

• Primary squamous cell carcinoma colon-a rare entity.
• Usually a pathological surprise.
• Very similar presentations like adeno counterpart.
• Metastasis from other sites must be ruled out.
• Treatment not standardized yet.

## 1 Introduction and importance

Worldwide, colorectal cancer is the second most common cancer in females and third most common in males. In India incidence is 4.3 and 3.4 per 100,000 in males and females respectively. Out of all malignancies of large bowel, adenocarcinoma is the most common accounting for more than 90% of cases [1]. Primary squamous cell carcinoma (SCC) of the colon is a rare entity and usually a pathological surprise, accounting for only 0.1–0.25/1000 of all colorectal malignancies [2]. Due to rarity of the disease and scarcity of data, its pathogenesis is not fully understood. There are different hypotheses described in literature regarding etiopathogenesis of primary SCC of colon. Chronic inflammatory and infective conditions of colon, radiation exposure and squamous differentiation of existing adenoma can give rise to SCC of colon. SCC of colon has a female preponderance in their 5th and 6th decade of life [2].It is impossible to differentiate SCC from adeno carcinoma clinically based on symptoms and signs as both have similar presentation. Primary SCC of colon is diagnosed by histopathological confirmation and the tumor should not be in continuity with the anal canal or any fistula lined track with the colon [3]. Currently, there are no standard treatment guidelines for this condition except complete excision along with lymphadenectomy as done in adenocarcinoma variety. In addition to surgery, chemotherapy may be administered either in Neo-adjuvant or Adjuvant setting depending upon the stage of the disease. It is reported that SCC of the colon has higher mortality rates than colonic adenocarcinoma [2]. We would like to share details of a patient presenting with symptoms suggestive of intestinal obstruction due to caecal mass which on histopathological examination found to be primary SCC. . This case report has been reported in line with the SCARE Criteria [4].

## 2 Case presentation

A, male patient in 6th decade, smoker without any known co-morbidities presented with chief complaints of abdominal pain, distension and obstipation for 5 days. He was suffering from intermittent abdominal pain since 9 months associated with malaise. Pain was confined to lower abdomen, insidious onset, dull aching and relieved on oral analgesics. There was no history of bleeding per rectum, malaena or hematemesis. There was history of weight loss of around 10 kg in this period with loss of appetite. Patient did not undergo any abdominal surgery or any treatment history for long duration in the past.There was no significant family history.

Clinical examination revealed pallor, abdominal distension without any tenderness on palpation. Routine blood investigations revealed hemoglobin of 4.4 gm/dl.Liver and kidney function tests are with in normal limit.

**Fig. 1:**
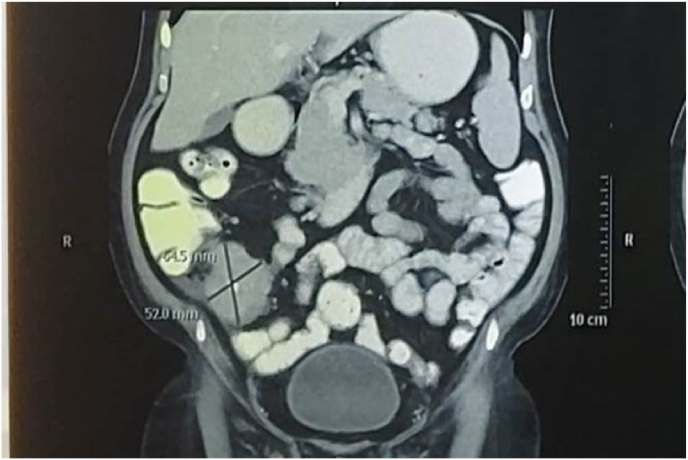
CT scan showing mass lesion in caecum (Sagittal section).

Erect X-ray of the abdomen showed multiple air fluid levels with dilated small bowel loops. Contrast enhanced computed tomography (CECT) of the abdomen revealed an enhancing soft tissue lesion in distal ileum/caecum with nearly complete luminal narrowing. There was dilation of small bowel loops proximal to the lesion (Fig. 1). Carcino Embryonic Antigen (CEA) level was 2.41ng/ml. In view of bowel obstruction and severe anaemia routine colonoscopy was not performed and he was taken up for emergency surgery after stabilization of his general condition.

Diagnostic laparoscopy followed by open radical right hemi-colectomy with ileo-transverse anastomosis was done. On examination of the specimen, there was an ulcero-proliferative lesion occupying whole lumen of the bowel. Postoperative period was uneventful. He was discharged on post operative day (POD7) in a stable condition.

**Fig. 2:**
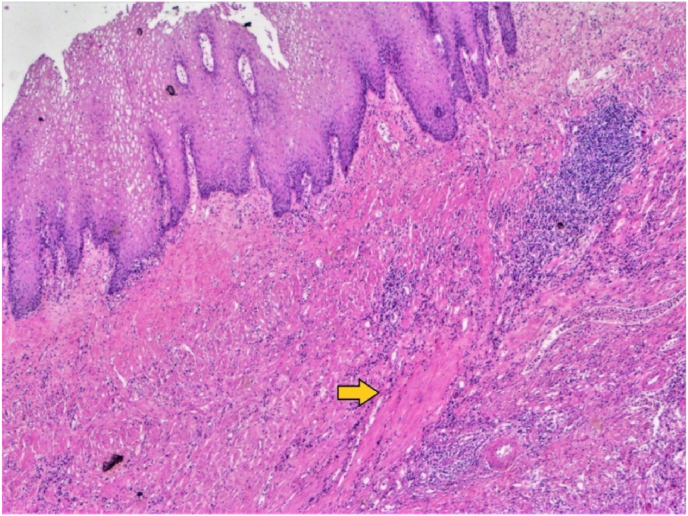
-H&E, 40x: Photomicrograph showing squamous metaplasia on the mucosal surface. The underlying muscularis propria (arrow) is infiltraed by chronic inflammatory infiltrate.

**Fig. 3:**
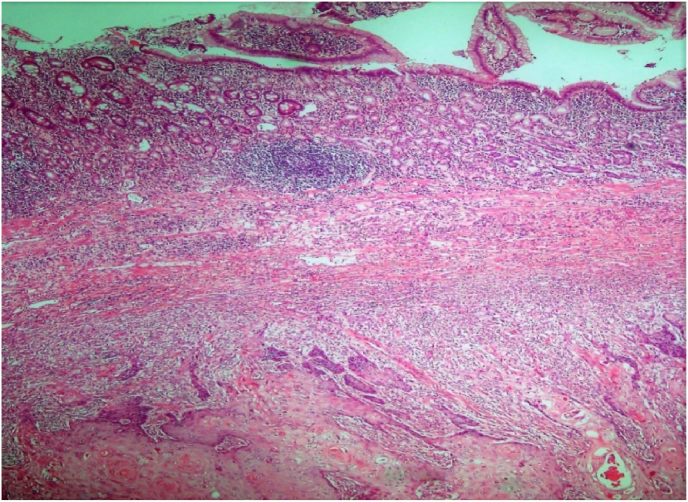
H&E, 40x: Photomicrograph shows uninvolved colonic mucosa (upper left), gastric metaplasia (upper right) and squamous cell carcinoma (bottom).

On histology, tumor was moderately differentiated squamous cell carcinoma and penetrating up to the sub serosal layer. There were no other tumor deposits and all the lymph nodes harvested were free from tumor spread (0/25). Details of the histological description described in the images below (Fig. 2, Fig. 3). According to AJCC tumor was p[T3 N0 Mx].To rule out any other primary site of SCC, whole body PET-CT was done 45 days after the index surgery which did not show any other FDG avid lesions in the body.Now he is on adjuvant chemotherapy and doing well.

## 3 Discussion

Primary colonic squamous cell carcinoma is extremely rare, representing less than 0.5% of all colorectal tumors, with an estimated incidence of 0.1% [5,6].In 1919 the first case of primary SCC of the colon was reported by Schmidtmann in a 65-year-old man [7]. Bhat et al. reported the first case of primary SCC of the colon in 1993 in a 55-year-old female from India [8]. Fewer than hundred cases and case reports has been described in literature since then [9]. On thorough review of literature we found 5–6 cases of primary SCC caecum, none of them presented as obstruction**.** Available data reveals that squamous cell carcinoma of the colon affects individuals with a mean age of 55–60 years. Women are more frequently predisposed to develop SCC than men, around 66% of cases occurred in women and 34% in men, [10,11]. Different hypothesis are formulated but, most accepted theory suggest origin either from a multi potent stem cell or from squamous metaplasia secondary to chronic irritation [6]. In our patient there was evidence of squamous metaplasia in the specimen (Fig. 2).Association of SCC of colon and chronic ulcerative colitis is around 1.7%, that favours the second hypothesis [11]. There are evidences of several case reports of SCC in patients with ulcerative colitis and also in patients with chronic infections including Schistosomiasis, Entamoebahistolytica and human papilloma virus (HPV) [12]. Change in bowel habits, constipation and bleeding per rectum can be presenting symptoms in both types [13]. Our patient presented with features of intestinal obstruction which can be a presenting feature of both adeno as well squamous cell carcinoma. Williams et al., in 1979 described that before the diagnosis of primary SCC of colorectum is made, certain criteria must be fulfilled. This criteria includes: (A) no evidence of SCC in any other part of body, so that chance of possible metastasis to colon should be ruled out; (B) in most of the cases of SCC of rectum, usually the epicenter is in the anal canal that extends in to the rectum. So there should be no evidence of anal canal SCC; (C) there should not be any colocutaneous fistula, because epithelial lined fistula tract can give rise to SCC and(D)lastly there should be histological confirmation of SCC [6]. In our patient whole body PET-CT was performed 45 days after the surgery. PET-CT did not show any other site of FDG avidity in the body. Our case fulfils all the criteria of a primary SCC of colon.

According to available data, surgery is the gold standard in primary SCC as is done in adenocarcinoma of colorectum. Some studies by Schneider et al. and Lafraniere et al. suggest addition of chemoradiotherapy to surgery as a useful option [14,15]. However, there is still no consensus on what would be an ideal treatment protocol for primary SCC of the colon. After a detailed discussion with medical oncologist, at present our patient is on chemotherapy.

In summary, our case was a 56 year male patient presented with features of intestinal obstruction with anaemia, abdominal pain, weight loss and malaise. After optimization curative surgery was performed. Postoperative period was uneventful. Histopathology was suggestive of SCC of colon, as discussed. Now patient is on adjuvant chemotherapy and he is doing well.

## 4 Conclusion

SCC colon is a rare entity. Radical excision with lymphadenectomy is the only curative option as per literature. Role of adjuvant therapy, needs to be addressed further. Before labelling as primary SCC, extension from anal canal SCC and metastasis from other primary site should be ruled out.

## Ethical approval

Not applicable.

## Sources of funding

Not applicable.

## Author contribution

Bhabani Sankar Sahoo: Research, Methodology, Formal review, Writing original draft, Guarantor. Sri Aurobindo Prasad Das: Supervision, Helped in patient management. Sonia Badwal: Provided histopathological slides and descriptions. Samiran Nundy: Chief supervisor. Final correction and approval of manuscript. Naimish N. Mehta: Chief supervisor. Final correction and approval of manuscript.

## Consent

Written informed consent was obtained from the patient for publication of this case report and accompanying images. A copy of the written consent is available for review by the editor-in- Chief of this journal on request.

## Registration of research studies

1. Name of the registry: Not applicable
2. Unique Identifying number or registration ID: Not applicable
3. Hyperlink to your specific registration (must be publicly accessible and will be checked): Not applicable

## Guarantor

Bhabani Sankar Sahoo, Sri Aurobindo Prasad Das, Sonia Badwal, Samiran Nundy, Naimish N. Mehta.

## Provenance and peer review

Not commissioned, externally peer-reviewed.

## Declaration of competing interest

No conflicts of interest.

## References

[1] Gordon P.L., Nivatvongs S. (2007).

[2] Linardoutsos D., Frountzas M., Feakins R.N., Patel N.H., Simanskaite V., Patel H. (2020). Primary colonic squamous cell carcinoma: a case report and review of the literature. Ann. R. Coll. Surg. Engl..

[3] Husain A., Kaundinya A.H., Hammed K.B., Sayed A. (2020). The surprise pathology-primary squamous cell carcinoma of the colon:a case report. Int. J. Surg. Case Rep..

[4] Agha R.A., Franchi T., Sohrabi C., Mathew G., for the SCARE Group (2020). The SCARE 2020 guideline: updating consensus surgical CAse REport (SCARE) guidelines. Int. J. Surg..

[5] Mondal S.K. (2011). Primary squamous cell carcinoma of the caecum: a casereport. J. Cancer Res. Therapeut..

[6] Michelassi F., Mishlove L.A., Stipa F., Block G.E. (1988). Squamous-cell carcinoma ofthe colon: experience at the University of Chicago, review of the literature, report of two cases. Dis. Colon Rectum.

[7] Williams G.T., Blackshaw A.J., Morson B.C. (1979). Squamous carcinoma of the colorectum and its genesis. J Pathol [Internet].

[8] Pigott J.P., Williams G.B. (1987). Primary squamous cell carcinoma of the colorectum:case report and literature review of a rare entity. J. Surg. Oncol..

[9] Gelas T., Peyrat P., Francois Y., Gerard P.J., Baulieux J., Gilly N.F. (2002). Primary squamous-cell carcinoma of the rectum: report of six cases and review of the literature. Dis Colon Rectum [Internet].

[10] Lyttle J.A. (1983). Primary squamous carcinoma of the proximal large bowel: report of a case and review of the literature. Dis. Colon Rectum.

[11] Vezeridis M.P., Herrera L.O., Lopez G.E. (1983). Squamous-cell carcinoma of thecolon and rectum. Dis. Colon Rectum.

[12] Sotlar K., Köveker G., Aepinus C., Selinka H.C., Kandolf R., Bültmann B. (2001). Humanpapillomavirus type 16-associated primary squamous cell carcinoma of therectum. Gastroenterology.

[13] Cabrera A., Pickren J.W. (1967). Squamous metaplasia and squamous cell carcinoma of the rectosigmoid. Dis. Colon Rectum.

[14] Lafreniere R., Ketcham A.S. (1985). Primary squamous carcinoma of the rectum. Report of a case and review of the literature. Dis. Colon Rectum.

[15] Schneider T.A., Birkett D.H., Vernava A.M. (1992). Primary adenosquamous and squamous cell carcinoma of the colon and rectum. Int. J. Colorectal Dis..

